# Magnetofected pollen gene delivery system could generate genetically modified *Cucumis sativus*

**DOI:** 10.1093/hr/uhae179

**Published:** 2024-06-27

**Authors:** Chan-Woo Park, Jun-Young Choi, Ye-Jin Son, Do-Hyeon Kim, Huanjun Li, Wanqi Liang, Chanhui Lee, Ki-Hong Jung, Yu-Jin Kim

**Affiliations:** Department of Life Science and Environmental Biochemistry, and Life and Industry Convergence Research Institute, Pusan National University, Miryang, 50463, Republic of Korea; Nongwoobio Nambu Breeding Institute, Miryang-si, 50414, Gyeongsangnam-do, Republic of Korea; Department of Life Science and Environmental Biochemistry, and Life and Industry Convergence Research Institute, Pusan National University, Miryang, 50463, Republic of Korea; Department of Life Science and Environmental Biochemistry, and Life and Industry Convergence Research Institute, Pusan National University, Miryang, 50463, Republic of Korea; Joint International Research Laboratory of Metabolic and Developmental Sciences, State Key Laboratory of Hybrid Rice, School of Life Sciences and Biotechnology, Shanghai Jiao Tong University, Shanghai 200240, China; Joint International Research Laboratory of Metabolic and Developmental Sciences, State Key Laboratory of Hybrid Rice, School of Life Sciences and Biotechnology, Shanghai Jiao Tong University, Shanghai 200240, China; Department of Plant and Environmental New Resources, Kyung Hee University, Yongin, 17104, Republic of Korea; Graduate School of Green Bio Science & Crop Biotech Institute, Kyung Hee University, Yongin, 17104, Republic of Korea; Department of Life Science and Environmental Biochemistry, and Life and Industry Convergence Research Institute, Pusan National University, Miryang, 50463, Republic of Korea

Dear Editor,

Horticultural crops, including vegetables and fruits, are cultivated in gardens or greenhouses as a part of the human diet. The Cucurbitaceae family, one of the largest vegetable families, comprises various plants such as cucumber (*Cucumis sativus* L.), watermelon (*Citrullus lanatus* Thunb.), zucchini (*Cucurbita pepo*), squash, and pumpkin (*Cucurbita* spp.). They are cultivated and consumed worldwide. However, owing to global warming, climate change, such as the increase in average annual temperatures and extreme weather events, is accelerating. These climate changes affect crop production both directly and indirectly [1].

**Figure 1:**
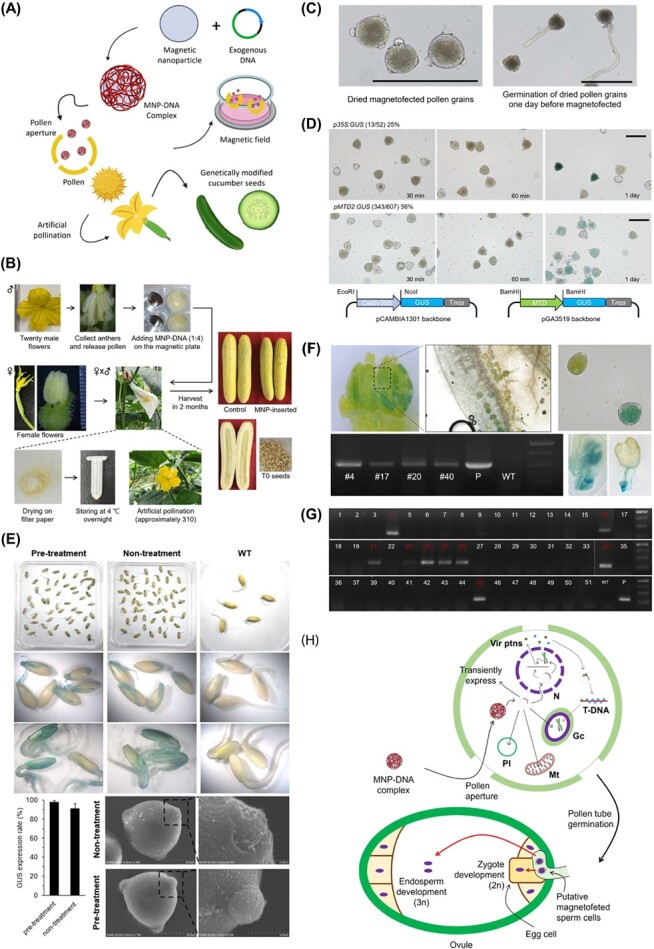
Magnetofected pollen gene delivery system applied to *C. sativus*. (A) Schematic illustration of pollen magnetofection. (B) Steps of production of magnetofected pollen gene delivered cucumber. 0.5 μg of MNPs (PolyMag200, Chemicell, Germany)) and 2 μg of plasmid DNA (200–250 ng/μg) were combined at a 1:4 ratio and allowed to stand for 15 minutes at room temperature for one magnetofection reaction. The MNP–DNA complexes were then added to a magnetofection buffer (15% sucrose, 0.01% boric acid, 1 mM Ca(NO3)2) containing pollen grains (Approximately 22 000 pollen grains, collected from 20 male flowers, were magnetofected with the MNP–DNA complexes. This corresponds to around 1100 pollen grains per male flower. [6]), and placed in a magnetic field (MagnetoFACTOR-24, Chemicell, Germany) for 30 minutes. Subsequently, the magnetofected pollen grains were carefully spread onto filter paper to remove the buffer and allowed to dry at room temperature overnight, followed by storage at 4°C. The next day, the magnetofected pollen grains were manually pollinated onto the stigma of female flowers. (C) Viability test of processed pollen. Dried magnetofected pollen grains were observed after the drying process and germination of magnetofected pollen grains which had dried 1 day before (Scale bar = 100 μm). (D) Increase in exogenous gene expression activity over time in the magnetofected pollen. Pollen-specific promoter (*OsMTD2* promoter) showed stronger GUS activity than the for constitutive promoter (Scale bar = 100 μm). (E) Statistical analysis of GUS expression was conducted with T1 seedlings (*n* > 130 seedlings for each group). Error bars represent the standard error of three repeats. No significant difference was observed between the presence and absence of treatment. Images of non-treated and pre-treated cucumber pollen grains were captured using scanning electron microscope. (Scale bar = 30 μm, 5 μm) The third row of the figure exhibits the cotyledon of the seedling. (F) Transgenic seeds were germinated and T1 seedlings were analyzed for GUS assay, DNA examination, and transferred to the soil. P is positive control, and wild type (WT) is not amplified for transgene, as negative control. (G) PCR analysis of T1 transgenic plants of Cas9 vector delivered experiments. P, pKIR1.1 plasmid; WT, wild type cucumber DNA. (H) Schematic illustration of putative model of DNA migration through pollen magnetofection. N, Vegetative nucleus; Gc, Generative cell; Mt, Mitochondria; Pl, Plastid.

Recently, an exogenous DNA delivery system using DNA-coated magnetic nanoparticles was developed to successfully generate transgenic cotton [2]. This technology is termed pollen magnetofection and uses positively charged Fe_3_O_4_ magnetic nanoparicles (MNPs) as exogenous DNA carriers to deliver them into the pollen aperture, which is a thinner pollen wall with high permeability, before pollination. When magnetofected pollen pollinates the stigma of female organs, exogenous DNA-transformed seeds can be obtained without regeneration, which is generally a barrier for tissue culture in *Agrobacterium*-mediated transformation [3]. However, it should be noted that this technique was not successful in other studies [4] on monocot plants. They investigated the effectiveness of pollen magnetofection in two monocot plants, maize and sorghum, but were unable to demonstrate any evidence of transient expression in the magnetofected pollen grains. However, another group [5] successfully generated transgenic maize with certain modifications. These studies highlight the challenges and limitations of applying pollen magnetofection to different plant species and suggest that further research and optimization are needed. Cucumber is an economically important crop that belongs to the family Cucurbitaceae. Here, we report the successful transformation of cucumber using an optimized pollen magnetofection method (Fig. 1A).

**Table 1:** Primers used in this study.

Primer name	Sequence (5′ to 3′)	Purpose
Cas1_F	TTCATCCAGCTCGTGCA	DNA certification
Cas1_R	GGCTTGATGAACTTGTAGAACT	DNA certification
Cas2_F	TTCATCCAGCTCGTGCA	DNA certification
Cas2_R	GGCTTGATGAACTTGTAGAACT	DNA certification
Hyg_F	GTGCTTGACATTGGGGAGTT	DNA certification
Hyg_R	GATGTTGGCGACCTCGTATT	DNA certification
CseIF4E_F	GAAGCCCAAGGGATAAAAGG	Genotyping
CseIF4E_R	TCTCTCCAGCCCTCACATTC	Genotyping
CsPDS_F	TCTCGGTTTCATTTCATCCA	Genotyping
CsPDS_R	CTGCCCCAGCAATCACTACT	Genotyping
eIF4E_sgRNA7_F	gattg CAAAACCCTAGAGGACGTGG	Gene cloning
eIF4E_sgRNA7_R	aaac CCGCCACGTCCTCTAGGGTTTTG	Gene cloning
eIF4E_sgRNA8_F	gattg AGGGTGAGGCTGATGAACTA	Gene cloning
eIF4E_sgRNA8_R	aaac CCTTAGTTCATCAGCCTCACCCT	Gene cloning
CsPDS_sgRNA1_F	gattg TCTCTGCTCTGAACTTGAGG	Gene cloning
CsPDS_sgRNA1_R	aaac CCTCAAGTTCAGAGCAGAGA	Gene cloning
AtU6_F	AGAAGAGAAGCAGGCCCATT	Gene cloning
gRNA_R	CGGTGCCACTTTTTCAAGTT	Gene cloning

In our study, we performed hand pollination by transferring transformed pollen grains onto the stigma of female cucumber flowers to produce transgenic seeds. Therefore, it is vital to ensure that pollen grains retain their viability before artificial pollination. However, after the pollen magnetofection procedure, it is necessary to remove and dry the magnetofection buffer, which shares the same components as the pollen germination medium, from the pollen grains to prevent the induction of pollen tube germination. To investigate whether pollen viability was affected by this process, cucumber pollen grains were observed immediately after the drying process of pollen magnetofection procedure (Fig. 1B). Dried pollen grains were intact and undamaged (Fig. 1C). The temporal dynamics of exogenous gene expression in magnetofected pollen grains were also investigated. The activity of the GUS reporter gene increased over time after its introduction (Fig. 1D), indicating successful delivery of the exogenous gene into pollen. These results demonstrate that exogenous genes can be transiently expressed in pollen grains and require a sufficient amount of time for their expression. To test the viability of stored pollen grains before artificial pollination, the dried pollen grains were kept in a 2 ml Eppendorf tube at 4°C overnight. After storage, the dried pollen grains successfully germinated in pollen germination media (Fig. 1C). These results demonstrate that pollen drying and storage did not significantly affect pollen viability or germination.

To test whether the promoter affected the delivery and expression of exogenous genes, we magnetofected cucumber pollen with two different GUS plasmid constructs (Fig. 1D). Each construct contained a different promoter for the *GUS* gene. In this study, our primary focus was on artificially pollinating pollen grains transformed with the *GUS* gene fused to the *Cauliflower mosaic virus* 35S promoter (p35S) to obtain transgenic seeds that ubiquitously express exogenous genes. The GUS expression efficiency was found to be higher in the *pMTD2:GUS* construct (56%), which is a strong pollen-specific promoter of *Oryza sativa* [7], compared to the *p35S:GUS* construct (26%). This observation highlights the importance of an appropriate promoter for driving gene expression in pollen grains to enhance the efficiency of transgenic plant production.

After artificial pollination with magnetofected pollen grains transformed with *p35S:GUS* construct, female flowers were immediately sealed with twist ties to prevent pollination. The fruits were then grown in an LMO field until they were fully ripened. Subsequently, the seeds were harvested and germinated for the GUS assay. GUS activity was successfully detected in the cotyledons and roots of T1 seedlings (Fig. 1E). The study conducted by Wang *et al*. [5] reported that removing the operculum of maize pollen was essential for pollen magnetofection. This is because pollen from cereal plants typically has only one aperture capped with an operculum [8], thereby blocking the entrance of materials. Although cucumber pollen has three apertures, we tested whether pollen pre-treatment affected the efficiency of the cucumber pollen. Based on these results (Fig. 1E), it was evident that pollen pre-treatment affected the apertures, causing them to open wider, but did not critically affect the transformation efficiency in cucumber plants. In addition, GUS expression was not only detected in the transgenic seedlings but also in the pollen grains of the T1 cucumber (Fig. 1F). However, not all pollen grains exhibited GUS expression, indicating that the transgenic cucumber plants were heterozygous. Zygotic embryos as T2 generation showed strong GUS staining which were collected from GUS-stained self-pollinated T1 cucumber fruits. This result showed the possibility of transgene transmission to the next generation by this method. Additionally, PCR analysis was conducted to analyze the heritability of exogenous genes in the next generation (Table 1). The integrated hygromycin-resistance gene in the GUS plasmid construct of the T1 cucumber genome was successfully amplified. However, we obtained about 10% DNA transmission by PCR analysis, less than GUS expression. MNP method does not use *Agrobacterium*; therefore, it is feasibility of transient transformation, as reported in maize [5] and lily [9]. Following the observation of decreasing activity of maize embryos over time, it was suggested that the GUS gene was diluted and gradually degraded as cells proliferated [5]. The reason of our observation that a relatively low ratio of gene integration in T1, despite obtaining a high ratio of GUS expression, is likely due to a low ratio of a complete integration of the vector into the host genome.

The clustered regularly interspaced short palindromic repeats/CRISPR-associated protein 9 (CRISPR/Cas9) system is the most widely used genome-editing technology in agriculture [10]. Using this optimized method, we successfully generated transgene delivered seeds using a CRISPR-Cas9 construct. However, we were only able to amplify the *Cas9* gene in less than 20% of the analyzed plants for all constructs (Fig. 1G), and genome editing in cucumbers has not yet been achieved.

With magnofected pollen, we hand-pollinated the female flower and obtained transgenic cucumber seeds up to 10%. However, many questions remain regarding how exogenous genes integrate into the host genome and are delivered to egg cells during double fertilization (Fig. 1H). While plasmid DNA is delivered into pollen, it can enter organelles or remain in the cytosol. As this method of transformation does not rely on *Agrobacterium*, how the T-DNA of the Ti plasmid is integrated into the host genome remains unknown. Once the Ti plasmid is transferred into the nucleus, it can be transcribed by the host RNA polymerase and translated. When T-DNA is translated, it can be integrated into the host genome, similar to *Agrobacterium*-mediated transformation. Alternatively, the plasmid could transiently express. However, these hypotheses must be confirmed through further experiments.
